# Where did the motor function of the cerebellum come from?

**DOI:** 10.1186/s40673-015-0029-8

**Published:** 2015-08-14

**Authors:** Marinella Coco, Vincenzo Perciavalle

**Affiliations:** Department of Biomedical and Biotechnological Sciences, Section of Physiology, University of Catania, Via Santa Sofia 64, 95125 Catania, Italy

**Keywords:** Cerebellum, Motor control, History

## Abstract

Until the end of 18th century, the role of the cerebellum remained obscure. The turning point occurred when Luigi Galvani showed that muscle contraction is due to electricity and Alessandro Volta produced the battery, an apparatus based on the pairing of silver and zinc plates separated by brine soaked paper disks, capable to generate electricity. Luigi Rolando, at beginning of 19th century, was impressed by these two observations. He thought that, since the brain generates the movement, it must contain a device generating electricity. As a battery, it should be formed by overlapping disks and the cerebellum for Rolando seemed to be the right structure for such a characteristic laminar organization. He argued that, if the cerebellum is the battery that produces electricity for muscle activity, its removal would produce paralysis. Consequently, Rolando removed the cerebellum in a young goat and observed that the animal, before dying, could no longer stand up. He concluded that the cerebellum is a motor structure as it generates the electricity which produces the movement. The conclusions of Rolando were criticized by Marie-Jean-Pierre Flourens who observed that animals undergoing cerebellectomy were still able to move, even if with problems of balance. Flourens concluded that the role of the cerebellum “is to put in order or to coordinate movements wanted by certain parts of the nervous system, excited by others”. It was necessary to wait up to 1891 when Luigi Luciani, observing a dog survived the cerebellectomy, described a triad of symptoms (asthenia, atony and astasis), unquestionably of cerebellar origin.

## Background

Who was the first to think that the cerebellum could play a motor role? In the Middle Ages, both in Europe and in the Islamic world, scholars believed that outer information from the external senses (touch, taste, smell, hearing and sight) was transferred to the brain to be combined into a unified perception, using a faculty called *common sense* or *inner sense* (for review, see Manzoni [1]).

This *inner sense* were believed to be housed not in the nervous tissue but in the ventricles of the brain (ventricular theory). It was believed that cerebral ventricles contained the *psychic pneuma* or *vital spirit* or *animal spirit*, a sort of special and light substance endowed with the power to perform sensory, motor and mental activities. The most widely accepted version of this theory was that the synthesized information from the all five senses was located in the front ventricle. Between the front ventricle and the middle ventricle was a storage space for representing previously perceived objects; the space was called the faculty of *imagination* or *representation*. The middle ventricle was believed to be involved in *cognition* and cognitions were thought to be transferred to the rear ventricle, under the cerebellum, for storage with the faculty of *memory* [1].

The ventricular theory was challenged from the early 16th century by several European scientists, although some remnants of this theory survived in medicine until the 18th century. In fact, up to the end of that century, the role of the brain structures within the posterior cranial fossa, cerebellum included, remained obscure.

Still in the early 1800s, Franz Joseph Gall (1758–1828), the creator of phrenology, argued that cerebellum is the area of self-preservation of the species [2].

## New knowledge

The turning point occurred thanks to two Italian scientists, Luigi Galvani and Alessandro Volta.

**Fig. 1:**
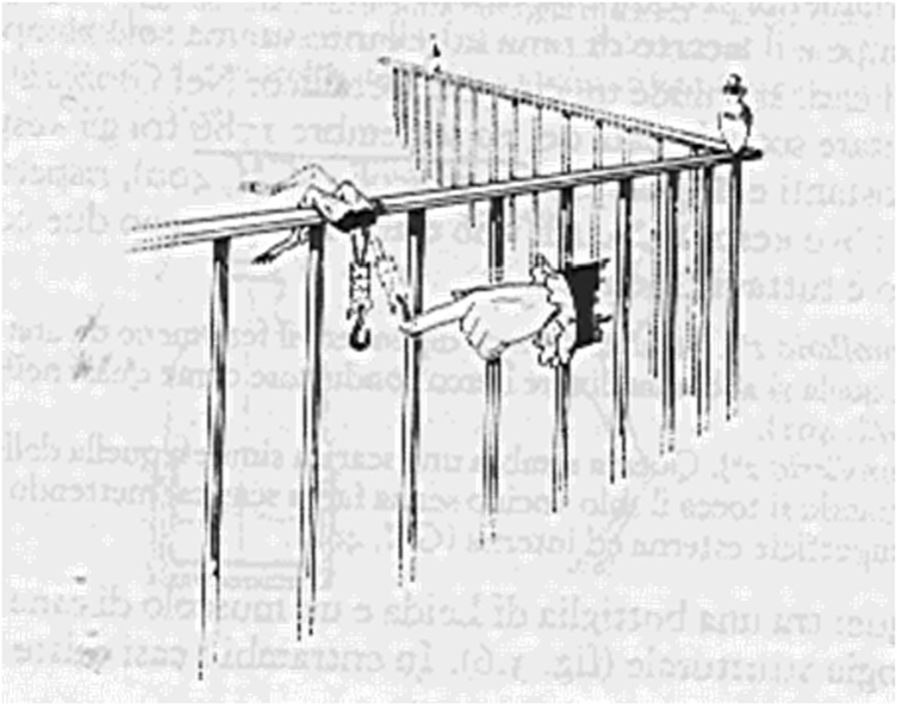
Luigi Galvani observed that the spasms of frog’s muscles occurred when he hooked the frog onto a metal railing (from http://ppp.unipv.it/Volta/Pages/ePage1.html)

Luigi Galvani (1737–1798), during the 1780’s, performed experiments at the University of Bologna involving electricity and frogs. He noticed that frogs’ legs hung from brass hooks on his metallic bannister twitched whenever the breeze made them knock against the ironwork (Fig. 1). Moreover, he observed contraction of the frog’s muscles when they were touched with a metallic rod at the moment when an electrostatic machine, in the laboratory, produced a discharge.

Galvani came to the conclusion that some kind of electricity, which he called *animal electricity*, was generated in the tissue of the frog and, flowing through the metal rod, activated the frog’s muscles. He thought of *animal electricity* as a fluid secreted by the brain, and proposed that flow of this fluid through the nerves activated the muscles. He grew convinced that the *vital spirit* was *animal electricity* flowing through the nerves and announced this to the Bologna Academy of Science in 1791 [3].

Alessandro Volta (1745–1827) was professor of experimental physics at the University of Pavia, from 1779 for almost 40 years. In 1792, Volta came to know of Galvani’s experiments on animal electricity. He initiated to repeat the experiments and at first his results agreed with those of Galvani. However, analyzing more closely the experimental conditions, Volta gradually became convinced that the contractions of the frog’s muscles were not due to the presence of electricity generated in the animal, but to some external electricity caused by the contact of the two metals. He concluded that different kinds of metals had electro-motive power at the point where they are in contact with the frog. He summarized his ideas with the expression: “It’s the difference in metals that does it”.

**Fig. 2:**
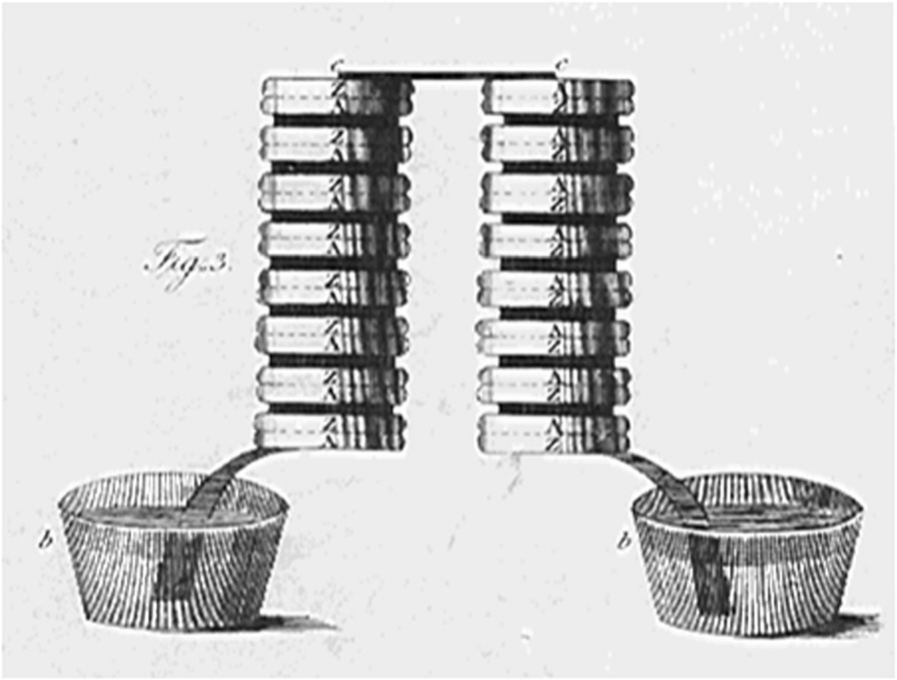
Column battery from the letter of Alessandro Volta to Sir Joseph Banks [4]

In late 1799, Volta produced the apparatus which made him famous: the battery, based on the pairing of silver and zinc plates separated by brine soaked paper disks (Fig. 2). This once more proved that bimetal contact was the real source of electrical power. Volta announced his invention to the scientific community on 20th March 1800 in a letter to Sir Joseph Banks, the President of the Royal Society in London [4].

## A new hypothesis

**Fig. 3:**
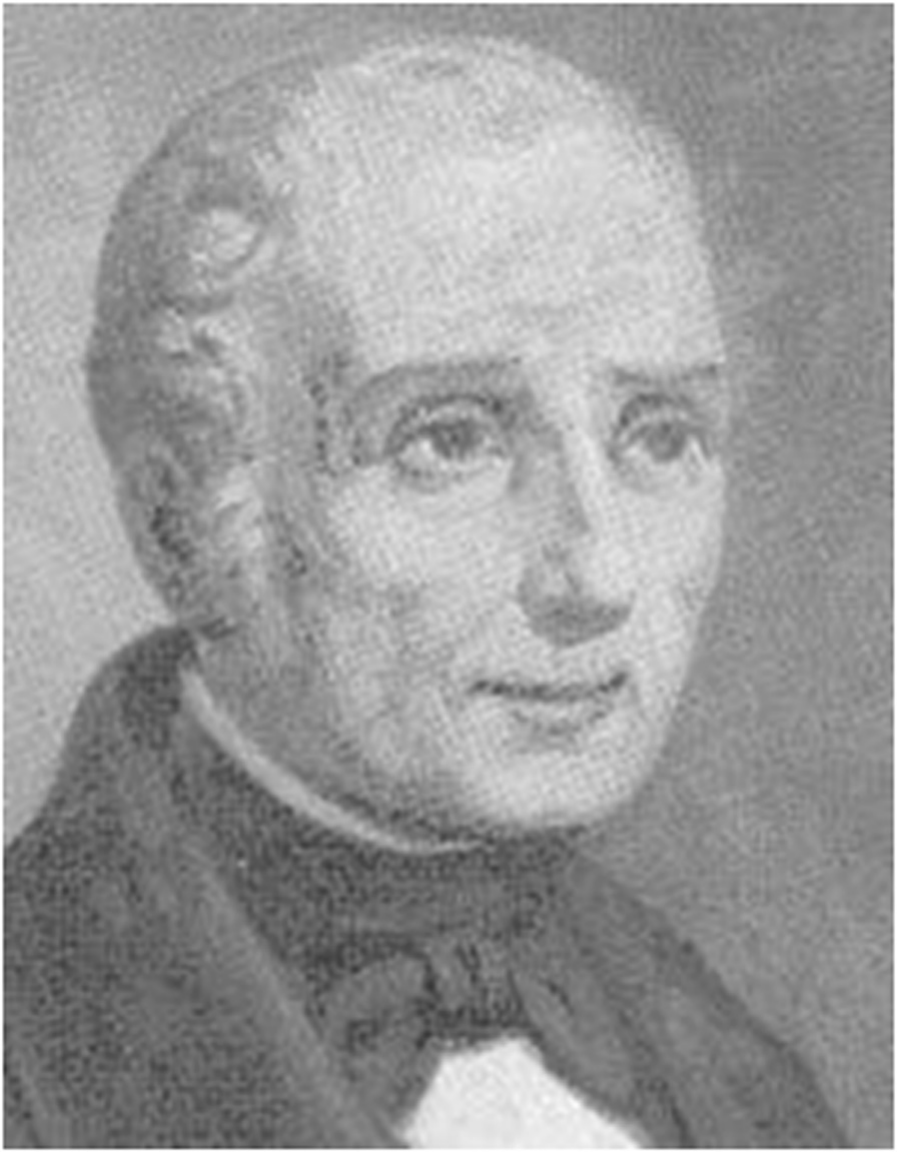
Luigi Rolando (particular of the paint of Pasquale Baroni in the Museum of Anatomy of the University of Turin)

It is at this moment that comes into play another Italian scientist, Luigi Rolando (1773–1831), who in 1804 became professor of physiology at the University of Sassari (Fig. 3).

Rolando was impressed by the two main observations of Galvani and Volta: muscle contraction is due to electricity and to generate electricity is necessary a battery. His reflection was the following: since the brain generates the movement, it must contain a device generating electricity. In his book published in 1809 [5], he writes “*se i fenomeni della locomozione sono l’effetto di un particolare meccanismo, questo non altrove che nell’encefalo andava ricercato*”. (if the phenomena of locomotion are the effect of a particular mechanism, this not elsewhere than in the encephalon had to be researched)

For Rolando this part of the brain, as a battery, should be formed by overlapping disks and the cerebellum seemed to be the right structure given its characteristic overlapping laminae, forming the so-called *arbor vitae*. Probably the term was coined by the Danish anatomist Jacob B. Winsløw (1669 –1760) for the similarity of cerebellar folia with the profile of leaves of the North American tree *Thuja occidentalis* or *Eastern Arborvitae*, introduced in France in 1534 by French explorers. It seems that the tree was named “*l’arbre de vie*” by the King Francis I [6] for analogy with the use of this expression in the Book of Proverbs, where the tree of life is associated with wisdom.

Rolando writes “*se dunque l’organo elettrico torpedinale e quelli del Siluro e del Ginnoto, fatti di sostanza albumino-gelatinoso-cartilaginea e simili attissimi sono a preparare, ed a sviluppare una quantità grandissima di fluido elettrico sufficiente per dare grandissime scosse, perché non potrà separarsi un principio consimile, quale si è il nerveo fluido dalle numerose lamine di sostanza midollare, giallognola, e cinerea del cervelletto? Quale maggiore evidenza potrassi desiderare per stabilire, che il cervelletto è un organo, la cui struttura è affatto consimile a quella dell’apparecchio del Volta?*” (if the electric organ of torpedo and those of wels catfish and electric eel, made of albuminous-gelatinous-cartilaginous substance, are perfectly suited to prepare and develop a large amount of electric fluid enough to give huge shocks, why a similar principle should not take place in form of nervous fluid from several sheets of yellowish and cinereous substance of the cerebellum? What greater evidence can be desired to establish that the cerebellum is an organ whose structure is absolutely similar to that of the Volta’s device?).

Rolando concluded that, if the cerebellum is the battery that generates electricity for muscle activity, its removal would produce paralysis. He writes “*Qual maggior prova per dimostrare, che dal suddetto viscere si separa un fluido analogo a quello, che dallo strumento citato si sviluppa? Qual più retta conseguenza, se esportato guasto o distrutto il cervelletto cessa ogni influsso del fluido nerveo nei muscoli destinati alla locomozione?*” (What most evidence to prove that the said organ generates a fluid similar to that which develops from the mentioned device? What most direct consequence if removed, destroyed or spoiled the cerebellum ceases any influence of the nervous fluid on the muscles for locomotion?).

Rolando, consequently, removed the cerebellum in a young goat and observed that the animal could no longer stand up “*non altrimenti che se fosse paralitico*” (not otherwise than if it was paralyzed). The animal survived for 24 h and died probably for postoperative sepsis.

Rolando concluded that the cerebellum is a motor structure as it generates the electricity which produces the movement.

## The criticisms

**Fig. 4:**
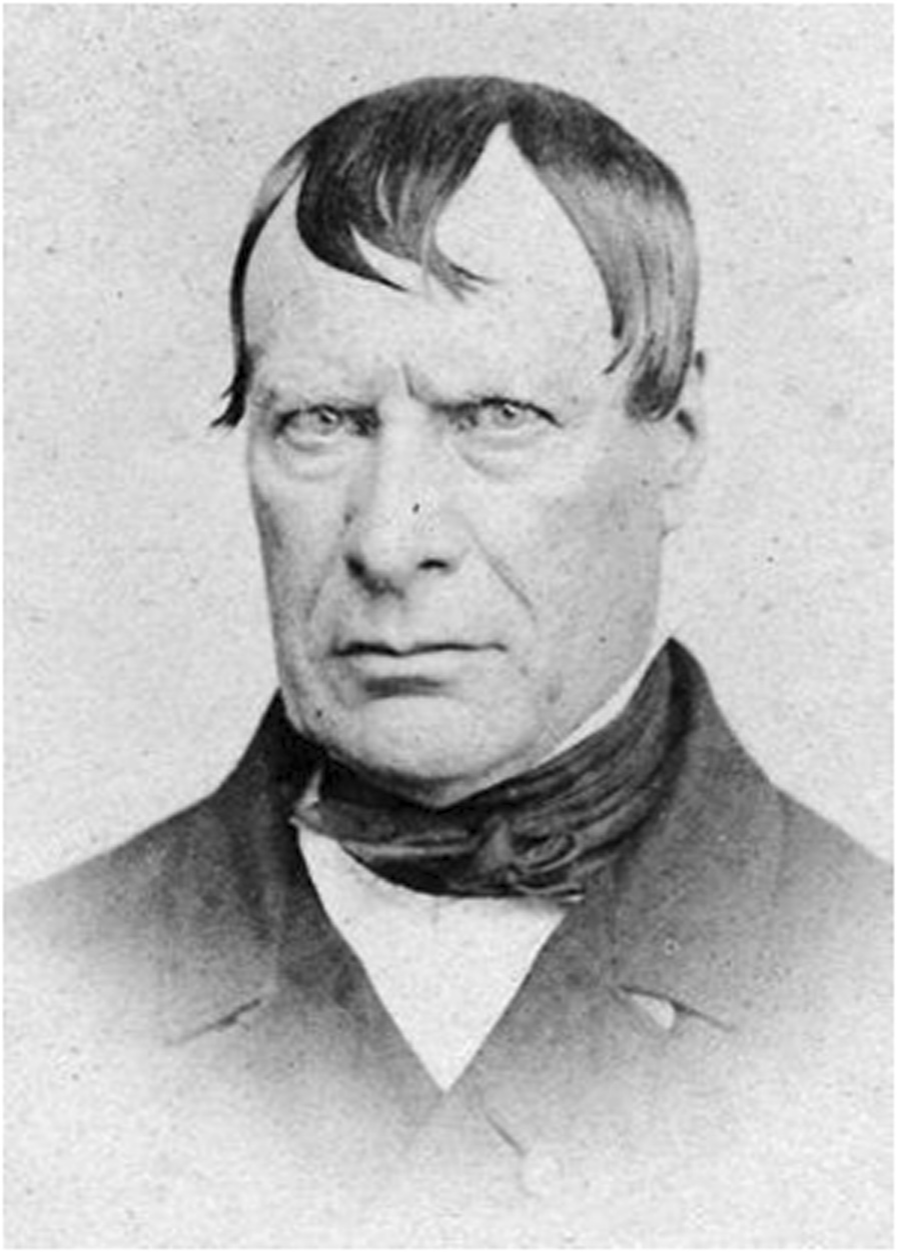
Marie-Jean-Pierre Flourens

The conclusions of Rolando were criticized by Marie-Jean-Pierre Flourens (1794–1867), professor of physiology at the Collège de France in Paris (Fig. 4). He observed that animals he submitted to cerebellectomy, with the intent to disprove the hypothesis of Gall, were still able to move, even if mating attempts failed for problems of balance. In his book published in 1824 [7], he concluded that “*dans le cervelet réside une propriété dont rien ne donnait encore l’idée en physiologie, et qui consiste a ordonner ou coordonner le mouvements voulus par certaines parties du système nerveux, excités par d’autres*” (in the cerebellum lies a property which nothing still gave the idea in physiology, that is to put in order or to coordinate movements wanted by certain parts of the nervous system, excited by others).

Since also its animals died shortly after the operation, Flourens hoped that the improvement of the surgery would allow to have animals surviving the cerebellectomy, to clearly distinguish the deficits due to the removal of cerebellum from those related to postoperative complications.

## Modern knowledge

**Fig. 5:**
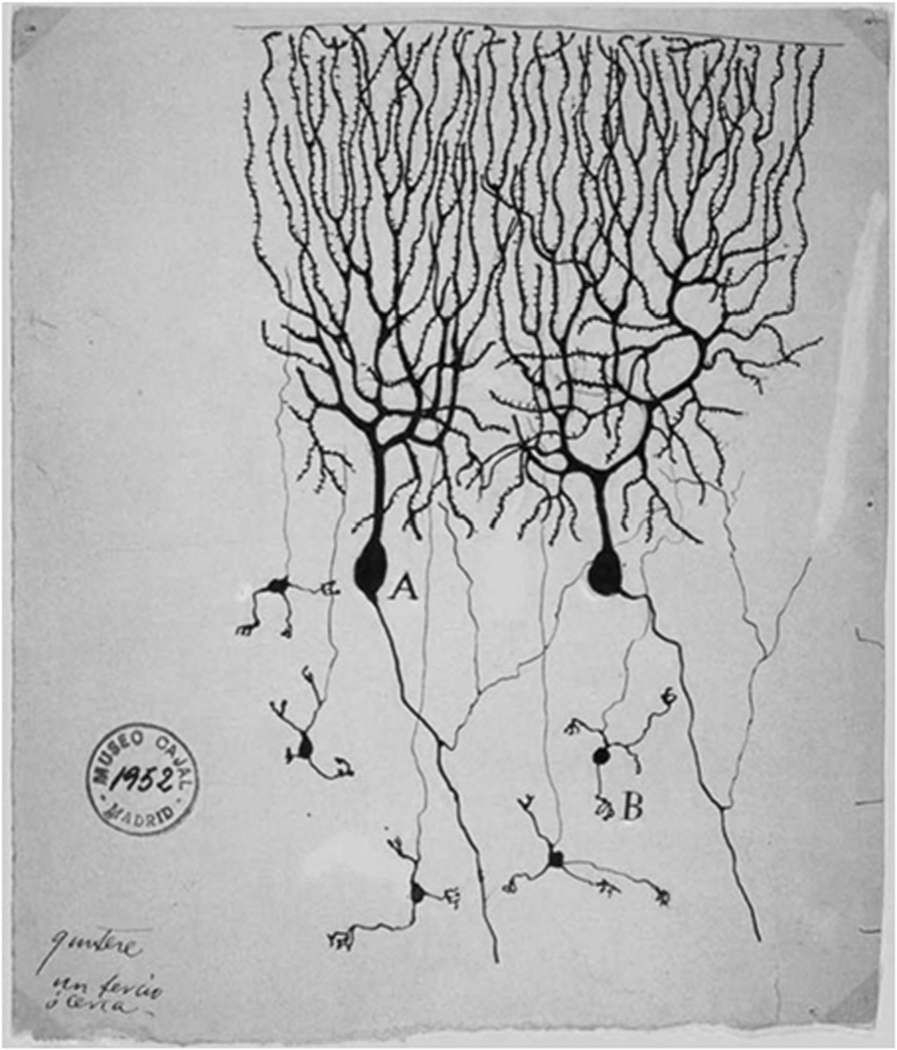
Drawing of Purkinje cells (**a**) and granule cells (**b**) from pigeon cerebellum by Santiago Ramón y Cajal. Instituto Santiago Ramón y Cajal, Madrid, Spain

It was necessary to wait the “germ theory of disease” of Louis Pasteur (1829–1895) and its application in clinical medicine, initially by Joseph Lister (1827–1912), with the use of carbolic acid as an antiseptic, and subsequently by Lawson Tait (1845–1899) and Ernst von Bergmann (1836–1907) which went from antisepsis to asepsis. These medical advances have allowed Luigi Luciani (1840–1919), in that period professor of physiology at the University of Florence, to publish in 1891 [8] his observations on a dog survived the cerebellectomy, with the description of a triad of symptoms (asthenia, atony and astasis), unquestionably of cerebellar origin, that confirmed the intuition of Flourens. In the same years (1894), Spanish neuroscientist and future Nobel laureate Santiago Ramón y Cajal (1852–1934) published what is considered the first modern textbook of neuroanatomy [9], with a clear depiction of the cerebellar cortex (Fig. 5).

The first systematic description of the symptoms of cerebellar lesions in man was carried out by the British neurologist Gordon Morgan Holmes (1876 –1965). During World War I he was neurologist with the British Expeditionary Forces and working in a field hospital he had the opportunity to investigate the effects of traumatic lesions involving the cerebellum. In 1922 Holmes’ observations on patients with cerebellar wounds as well as tumors were published in his Croonian Lectures to the Royal College of Physicians [10].

The general conclusion reached before World War II was that the main role of the cerebellum is to detail the different aspects of a movement, not to initiate movements or to decide which movements to execute. After the war, there was a significant increase in knowledge of circuitry and electrophysiology of the cerebellum, summarized in 1967 in a book, *The Cerebellum as a Neuronal Machine* [11], written by the Nobel laureate John C. Eccles (1903–1997), Japanese neuroscientist Masao Ito, and Hungarian anatomist János Szentágothai (1912–1994), followed in 1974 by a review, *Cerebrocerebellar communication systems* [12], written by two neurophysiologists, the American Gary I. Allen and the Japanese Nakaakira Tsukahara (1933–1985).

In the same years it was suggested that the cerebellum is involved in motor learning. Most theories that attempt to explain the role of cerebellar circuits in motor learning are derived from the ideas of British neuroscientist and psychologist David C. Marr (1945–1980) and of American engineer James S. Albus (1935–2011). Both attributed an important role to climbing fiber activity capable to cause synchronously activated parallel fiber inputs, to be strengthened for Marr [13] and to be weakened for Albus [14]. In the 1980s, the discovery in the cerebellum of Long Term Depression (LTD) was considered as a form of synaptic plasticity involved in motor learning. LTD occurs when impulses of a set of granule cells and one climbing fiber reach the same Purkinje cell synchronously and repeatedly; synaptic transmission from the granule cells to the Purkinje cell is then persistently depressed [15]. Although LTD is now well characterized, its contribution to motor learning remain controversial [16].

Up to the 1990s the cerebellum was almost universally believed to be primarily involved in movement, but latest results have led to consider that view too restrictive. Imaging studies have allowed to detect cerebellar activation in relation to cognitive activities and numerous correlations between the cerebellum and non-motor regions of the cerebral cortex were highlighted. Moreover, in patients with lesions restricted to the cerebellum, non-motor symptoms have been frequently recognized. In 1998, the American neurologist Jeremy D. Schmahmann [17] described the *Cerebellar Cognitive Affective Syndrome*, characterized by impairment of executive functions, difficulties with spatial cognition, personality change and language deficits. Table 1 summarizes the major contributions to the current knowledge of the cerebellum.

**Table 1:** Major contributions to the current knowledge of the cerebellum

Year	Author	Contribution
1809	Luigi Rolando	The cerebellum is the battery that produces the electricity necessary for generating muscular contraction
1824	Marie-Jean-Pierre Flourens	The role of the cerebellum is not that of generating the movement but to regulate it
1891	Luigi Luciani	Description, in a dog survived the cerebellectomy, of a triad of symptoms (asthenia, atony and astasis) unquestionably of cerebellar origin
1894	Santiago Ramón y Cajal	Publication of the first modern textbook of neuroanatomy with a clear description of the cerebellar cortex.
1922	Gordon Morgan Holmes	Systematic description of the symptoms of cerebellar lesions in man
1967	John C. Eccles, Masao Ito, and János Szentágothai	Book: The Cerebellum as a Neuronal Machine
1969	David C. Marr	Hypothesis about cerebellum and motor learning: A theory of cerebellar cortex
1971	James S. Albus	Hypothesis about cerebellum and motor learning: A theory of cerebellar function
1974	Gary I. Allen and Nakaakira Tsukahara	Review: Cerebrocerebellar communication systems
1982	Masao Ito and Masanobu Kano	Description in the cerebellum of the Long Term Depression
1998	Jeremy D. Schmahmann	Description of the Cerebellar Cognitive Affective Syndrome

## Conclusion

Luigi Rolando devoted his life to the study of the brain. Despite his outlandish theory on the cerebellum, he provided a major contribution to the advancement of neurosciences and many neural entities are named after him: the *substantia gelatinosa of Rolando* in the spinal cord, the *fissure of Rolando* or central sulcus, the *Rolandic operculum* or post-central operculum, the *Rolandic artery* or central sulcal artery, the *Rolandic vein* i.e., the vein posterior to Trolard’s vein draining the parietal lobe, the *pre-Rolandic artery* or precentral sulcal artery, and the *Rolandic epilepsy* or benign childhood epilepsy with centrotemporal spikes (BCECTS), the most common epilepsy syndrome in childhood.
